# Angiotensin-Converting Enzyme Inhibitor (ACEI)-Induced Angioedema After Re-exposure to Lisinopril: A Case Report and Clinical Review

**DOI:** 10.7759/cureus.89784

**Published:** 2025-08-11

**Authors:** Leila C Tou, Jacob D DeMott, José M Palacios

**Affiliations:** 1 Department of Internal Medicine, Wright State University Boonshoft School of Medicine, Dayton, USA

**Keywords:** ace inhibitor induced angioedema, angiotensin converting enzyme inhibitors, bradykinin mediated angioedema, drug-induced angioedema, side effects of lisinopril

## Abstract

Angiotensin-converting enzyme inhibitor (ACEI)-induced angioedema is an uncommon but potentially life-threatening adverse effect that can occur unpredictably, even after prolonged use. Prompt recognition and appropriate management are essential. We present the case of a 54-year-old Caucasian male with a history of ACEI-induced angioedema who developed isolated lingual swelling after re-exposure to lisinopril. His symptoms were unresponsive to antihistamines, corticosteroids, and epinephrine but resolved with supportive care following ACEI discontinuation. This case underscores the risk of recurrent angioedema following ACEI re-exposure and reinforces the recommendation for lifelong avoidance of ACEIs in patients with a prior diagnosis of ACEI-induced angioedema. Angiotensin receptor blockers (ARBs), which carry a lower risk of angioedema, may serve as preferred alternatives in high-risk patients. Patient education remains critical to preventing recurrence.

## Introduction

Since the development of the first angiotensin-converting enzyme inhibitor (ACEI), captopril, in 1975, ACEIs have become among the most prescribed antihypertensives, with more than 40 million patients worldwide currently receiving these medications [1-2]. Lisinopril remains the most prescribed ACEI in the United States [2]. Beyond their antihypertensive effects, ACEIs play a crucial role in managing coronary artery disease, chronic heart failure, diabetes, and chronic kidney disease [3].

While generally well tolerated, ACEIs are associated with several adverse effects, including hyperkalemia, acute renal failure, hypotension, and cough [4]. Among these, angioedema is a rare yet well-documented adverse effect [1-3]. Angioedema is characterized by localized swelling of the skin, subcutaneous tissue, and/or mucous membranes, which can be potentially life-threatening if the airway is involved [1,5]. The incidence of angioedema is estimated to be between 0.1% and 0.7% in patients taking ACEIs [5]. With rising ACEI use, angioedema-related hospitalizations have increased from 3.3 to 4.0 per 100,000 admissions over 15 years [1].

Despite this, ACEI-induced angioedema can be challenging to diagnose due to several factors, including the lack of specific diagnostic tests, the delayed and unpredictable onset of symptoms, and the potential to misinterpret symptoms as other conditions such as histamine-mediated angioedema [6].

In this report, we describe a case of angioedema in a middle-aged Caucasian male with a history of ACEI-induced angioedema who presented with similar features after ACEI re-exposure. This case highlights the risk of recurrent angioedema following ACEI re-exposure and emphasizes the importance of patient education for the permanent discontinuation of this drug class. It also raises the question of whether angiotensin receptor blockers (ARBs) should be favored initially, especially in high-risk populations.

## Case presentation

A 54-year-old Caucasian male with a history of ACEI-induced angioedema, coronary artery disease, hypertension, hyperlipidemia, and tobacco use presented with progressive left-sided tongue swelling over four hours. He had been taking his spouse’s lisinopril for two weeks after running out of his prescribed irbesartan. He noticed speech difficulties secondary to the swelling but denied cough, hoarseness, shortness of breath, dysphagia, rash, or itching. He attempted diphenhydramine at home without improvement. He denied exposure to new foods, insect bites, or trauma. There was no history of allergies, asthma, atopy, or family history of angioedema.

The patient reported a similar episode of right-sided cheek swelling without airway compromise four years earlier, which began approximately one year after starting lisinopril. He denied other adverse reactions to lisinopril, including cough. At that time, he was diagnosed with ACEI-induced angioedema, which resolved after discontinuation of lisinopril. After this episode of angioedema, the patient was switched to irbesartan without recurrence of symptoms.

**Figure 1:**
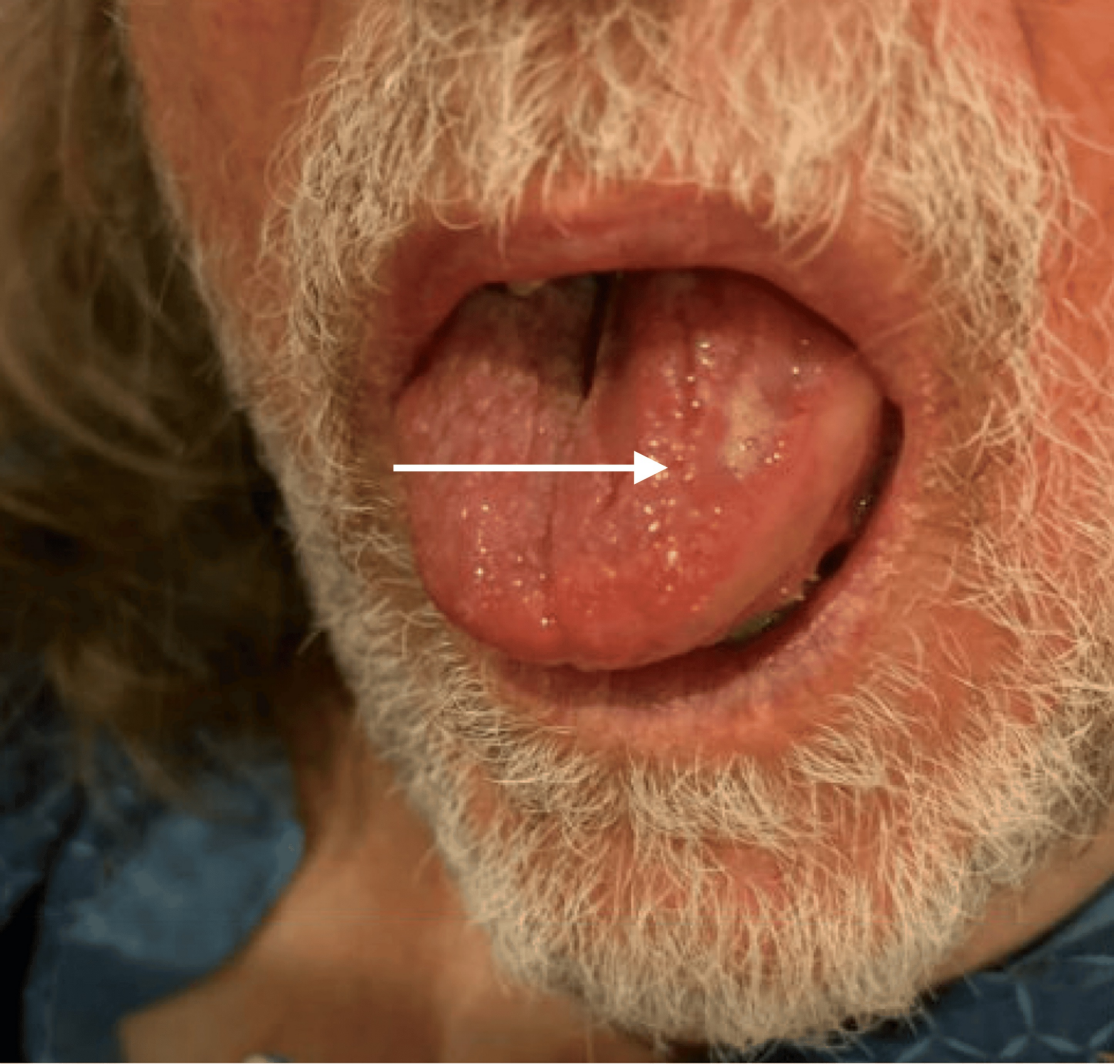
Left-sided tongue swelling on initial presentation, without evidence of respiratory compromise.

On presentation to the emergency department, he was afebrile and hemodynamically stable, with a temperature of 97.6 degrees Fahrenheit, blood pressure of 129/71, pulse of 75 beats per minute, respiratory rate of 16 breaths per minute, and an oxygen saturation of 98% on room air. Physical examination revealed left-sided lingual swelling with a patent airway and no signs of respiratory distress (Figure 1). Examination of the oropharynx showed no posterior pharyngeal edema, uvular deviation, or stridor. There was no swelling of the lips, face, or periorbital region. The neck was supple, with the trachea in the midline, no cervical lymphadenopathy, and no tenderness or crepitus. Cardiopulmonary examination was unremarkable, with clear breath sounds bilaterally and normal heart sounds. Skin examination revealed no rash or urticaria. Laboratory evaluation, including complete blood count and basic metabolic panel, was unremarkable (Table 1), as were ECG and chest X-ray. He received tranexamic acid, methylprednisolone, famotidine, epinephrine, and diphenhydramine with minimal clinical improvement.

**Table 1:** Outline of laboratory investigations.

Test	Result	Normal value
Complete blood count		
White blood cell count (K/uL)	7.8	3.5-10.9
Hemoglobin (g/dL)	13.2	13.0-17.7
Hematocrit (%)	39.7	37.5-51.0
Platelet Count (K/uL)	218	150-400
Basic metabolic panel		
Sodium (mEq/L)	141	135-148
Potassium (mEq/L)	4.2	3.4-5.3
Chloride (mEq/L)	107	96-110
Carbon dioxide (mEq/L)	23	19-32
Glucose (mEq/L)	98	70-99
Blood urea nitrogen (mg/dL)	11	3.0-29.0
Creatinine (mg/dL)	0.8	0.5-1.4

Given the timing of symptom onset, prior history of angioedema, and exclusion of other potential causes, the Naranjo probability scale validated tool for assessing the likelihood of adverse drug reactions indicated a *definite* causal relationship between lisinopril and the angioedema [7]. He was admitted to the critical care unit for close airway monitoring. Overnight, his lingual swelling and speech improved significantly. He was discharged the next day on irbesartan. At outpatient follow-up, the patient reported complete resolution of symptoms, and his blood pressure remained well controlled on irbesartan.

## Discussion

ACEI-induced angioedema is a form of bradykinin-mediated angioedema [3,5]. Because angiotensin-converting enzyme (ACE) is a major enzyme responsible for bradykinin catabolism, ACEIs promote angioedema through the accumulation of vasoactive peptides such as bradykinin and substance P [6,8]. Angioedema can affect the face, lips, oral cavity, oropharynx, gastrointestinal tract, and genitalia [5-6,8], and is particularly dangerous when it involves the upper airway [5-6,9]. Prompt recognition is critical for patient survival [5].

Differentiating bradykinin-mediated angioedema from histamine-mediated forms is essential for guiding management [6,9]. Although no validated biomarkers currently exist, several clinical features can aid diagnosis [6,8]. Unlike histamine-mediated angioedema, bradykinin-mediated angioedema typically does not present with urticaria and often has a delayed onset [6]. Histamine-mediated angioedema usually occurs within one hour of allergen exposure, whereas bradykinin-mediated cases may appear days to years after starting an ACEI [6]. Only 18% of patients develop symptoms within the first month of ACEI therapy, with a mean onset of 12 months [8]. Our patient developed symptoms initially after one year of ACEI use and again after one week of re-exposure, highlighting the unpredictable and delayed nature of onset.

Given the bradykinin-driven mechanism, patients with ACEI-induced angioedema typically do not respond to steroids, antihistamines, or epinephrine, as seen in our patient [8]. Despite this, these therapies are often administered empirically, especially when the diagnosis is uncertain. A poor response to antihistamines and steroids supports a diagnosis of bradykinin-mediated angioedema rather than an allergic etiology [8].

Management primarily involves immediate discontinuation of the offending ACEI and vigilant airway monitoring, with readiness for intubation or surgical airway if necessary [5]. Patients diagnosed with ACEI-induced angioedema require permanent discontinuation of this drug class to prevent recurrence [2]. This case underscores the importance of counseling patients with ACEI-induced angioedema to avoid future ACEI use. Clear patient education at diagnosis is essential to prevent inadvertent re-exposure, especially since patients may not recognize ACEIs by name or recall prior reactions.

Although no therapies are currently approved by the U.S. Food and Drug Administration (FDA) specifically for ACEI-induced angioedema, off-label treatments used in hereditary angioedema, such as plasma-derived C1 esterase inhibitors (C1-INH), which replace deficient enzyme activity, and icatibant, a selective bradykinin B2 receptor antagonist, have been employed [1,2,8]. However, evidence supporting their efficacy in ACEI-induced angioedema remains limited and primarily derived from small observational studies [8]. Tranexamic acid, an antifibrinolytic agent that may reduce bradykinin production, can also be used off-label, with limited observational data supporting its use [10]. While not endorsed by guidelines for acute management, tranexamic acid may be considered in settings where targeted bradykinin therapies are unavailable or cost-prohibitive [10].

The incidence of ACEI-induced angioedema is expected to rise with increased use of combination drugs (e.g., ACEI with hydrochlorothiazide), which may increase patient exposure [1]. In the Omapatrilat Cardiovascular Treatment Assessment Versus Enalapril (OCTAVE) trial, several risk factors were identified, including Black race, age over 40 years, smoking, seasonal allergies, female sex, history of drug rash, and use of immunosuppressive therapy [11]. In high-risk patients, alternative therapies such as angiotensin II receptor blockers (ARBs) should be preferred [11]. Given the significantly lower incidence of angioedema with ARBs [1,4] and their comparable cardiovascular and renal benefits [4,12], initiating ARBs over ACEIs in patients at higher risk for angioedema may be reasonable. However, such decisions should be individualized and balanced with guideline-based recommendations.

## Conclusions

ACEI-induced angioedema is an uncommon but potentially life-threatening adverse reaction that may occur unpredictably, even after prolonged use or prior tolerance. This case illustrates the risk of recurrence following re-exposure and underscores the importance of prompt recognition and supportive management. Clinicians must educate patients about the risks associated with ACEIs and ensure clear documentation and communication to prevent inadvertent re-exposure. In high-risk individuals or those with a history of angioedema, ARBs may offer a safer alternative, particularly for patients with underlying cardiovascular or renal disease. Enhanced awareness and patient education are essential to preventing recurrence and ensuring the safe long-term management of chronic conditions.
